# New Volume

**Published:** 1873-01-01

**Authors:** 


### Medical Examiner.

*A Semi-Monthly Journal of Medical Sciences.*

EDITED BY

N. S. DAVIS, M. D., AND F. H. DAVIS, M. D.

Chicago, January 1st, 1873.

### EDITORIAL.

### NEW VOLUME.

The present number commences the fourteenth volume of The Medical Examiner, and through its columns we wish all our readers *a happy New Year.* It seems but as yesterday when we issued the first number, and yet thirteen years of active, earnest life have passed, during which we have given a small portion of almost every day to the work of furnishing our readers with something calculated to aid them in the great and responsible duty of preventing, alleviating and curing disease. To aid in diffusing a knowledge of whatever we found of special value, either in our own practice or in the current literature of the day, and to encourage improvement in the social and educational interests of the profession, have been the controlling objects of our editorial work.

One year since we altered the form of The Examiner, doubling its columns and increasing the frequency of its issue, thereby giving our readers a much larger amount of matter in the year, and giving it on excellent type and paper. This, of course, added to the expense of publication. The improvements already made will be continued, and others will be made from time to time, as opportunity offers. We have seldom said anything in these columns about pecuniary matters, but have waited patiently for each subscriber to be actuated by his own sense of duty. And all we wish to say now is, that this is the time for every reader to renew his subscription. Three dollars is a small sum, and easily paid at the beginning of each year. Send it along, with as many contributions for our columns as you can, and you will thereby make the coming year more happy and more useful than the past.

